# Midwives’ experiences of cultural competency training and providing perinatal care for migrant women a mixed methods study: Operational Refugee and Migrant Maternal Approach (ORAMMA) project

**DOI:** 10.1186/s12884-021-03799-1

**Published:** 2021-04-29

**Authors:** Frankie Fair, Hora Soltani, Liselotte Raben, Yvonne van Streun, Eirini Sioti, Maria Papadakaki, Catherine Burke, Helen Watson, Mervi Jokinen, Eleanor Shaw, Elena Triantafyllou, Maria van den Muijsenbergh, Victoria Vivilaki

**Affiliations:** 1 College of Health, Wellbeing and Life Sciences, Sheffield Hallam University, 34 Collegiate Cres, Sheffield, S10 2BP UK; 2 Department of Primary and Community Care, Radboud University Medical Centre, Nijmegen, Netherlands; 3 Department of Midwifery, Faculty of Health and Caring Sciences, University of West Attica, Athens, Greece; 4 Department of Social Work, School of Health Sciences, Hellenic Mediterranean University, Heraklion, Greece; 5 Practice and Standards Professional Advisor, The Royal College of Midwives, London, UK; 6 President of European Midwives Association (EMA), Antwerpen, Belgium; 7 Vice Chair European Forum for National Nurses and Midwives Associations (EFNNMA), Lisbon, Portugal; 8 Centre for the History of Science, Technology and Medicine at the University of Manchester, Manchester, UK; 9 Pharos, Centre of Expertise on Health Disparities, Utrecht, Netherlands

**Keywords:** Transients and migrants, Cultural competency, Staff development, Maternal health service, Perinatal care, Midwifery, ORAMMA

## Abstract

**Background:**

The number of international migrants continues to increase worldwide. Depending on their country of origin and migration experience, migrants may be at greater risk of maternal and neonatal morbidity and mortality. Having compassionate and culturally competent healthcare providers is essential to optimise perinatal care. The “Operational Refugee and Migrant Maternal Approach” (ORAMMA) project developed cultural competence training for health professionals to aid with providing perinatal care for migrant women. This presents an evaluation of ORAMMA training and explores midwives’ experiences of the training and providing care within the ORAMMA project.

**Methods:**

Cultural competence was assessed before and after midwives (*n* = 35) received ORAMMA compassionate and culturally sensitive maternity care training in three different European countries. Semi-structured interviews (*n* = 12) explored midwives’ experiences of the training and of caring for migrant women within the ORAMMA project.

**Results:**

A significant improvement of the median score pre to post-test was observed for midwives’ knowledge (17 to 20, *p* < 0.001), skills (5 to 6, *p* = 0.002) and self-perceived cultural competence (27 to 29, *p* = 0.010).

Exploration of midwives’ experiences of the training revealed themes of “appropriate and applicable”, “made a difference” and “training gaps” and data from ORAMMA project experiences identified three further themes; “supportive care”, “working alongside peer supporters” and “challenges faced”.

**Conclusions:**

The training improved midwives’ knowledge and self-perceived cultural competence in three European countries with differing contexts and workforce provision. A positive experience of ORAMMA care model was expressed by midwives, however clearer expectations of peer supporters’ roles and more time within appointments to assess the psychosocial needs of migrant women were desired. Future large-scale research is required to assess the long-term impact of the ORAMMA model and training on practice and clinical perinatal outcomes.

**Supplementary Information:**

The online version contains supplementary material available at 10.1186/s12884-021-03799-1.

## Background

The number of international migrants continues to increase worldwide [1]. Within the World Health Organization (WHO) European region almost 10% of the population are international migrants and many are women of reproductive age [2]. Consequently, European maternity care systems are challenged with the provision of good quality care tailored to female migrants’ needs.

Depending on their country of origin and migration experience, migrants may be at greater risk of maternal and neonatal morbidity and mortality compared to native women [3–5]. Unfamiliarity with the host country’s maternity care, language barriers and maternity care insufficiently tailored to the cultural, medical and social needs of this population contribute to poorer outcomes [3, 6–8].

Diversity in language, health literacy, culture and religion as well as socioeconomic position amongst migrants requires consideration by healthcare providers in order to deliver effective person-centred care. To meet migrant women’s needs, healthcare systems that provide appropriate care through compassionate and culturally competent healthcare providers are essential [6, 9, 10]; where ‘cultural competence’ is defined as “*the attitudes, knowledge and skills necessary to deliver high quality care to ethnically and culturally diverse patient populations*” [11]. Reviews have shown cultural competence training can improve healthcare providers’ cultural competence in general [12–14]. Training on caring for asylum-seeking and refugee women has previously been developed for midwives [15]; however, to our knowledge no study has assessed the effectiveness of training on maternity care providers’ cultural competence, nor compared the impact of training in different countries. A recent systematic review of migrant women’s experiences of maternity care, with studies undertaken within 14 different European countries highlighted a need for healthcare professionals to receive training in culturally competent care [16]. Therefore as part of a larger project, “Operational Refugee and Migrant Maternal Approach” (ORAMMA), we developed and assessed the impact of culturally sensitive maternity care training for midwives in three European countries (Greece, the Netherlands and United Kingdom (UK)) with different migration histories and healthcare organisation.

**Fig. 1:**
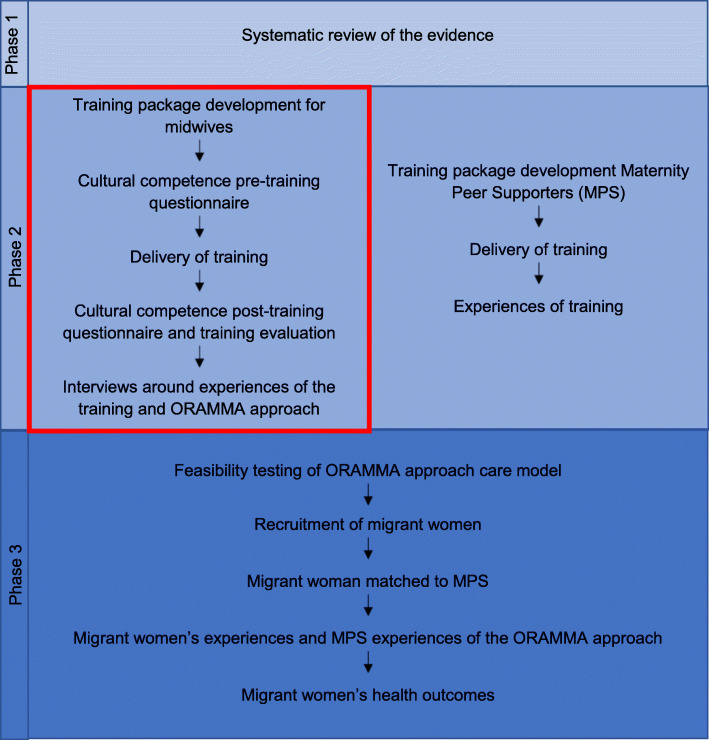
Schematic overview of the ORAMMA project phases

ORAMMA aimed to develop and implement an evidence-based approach for migrant maternity care to improve pregnancy outcomes. The project involved three phases: i) systematic review of current evidence to inform and design the ORAMMA integrated care model including midwife-led continuity of care and integration of Maternity Peer Supporters (MPS) [17] ii) developing and delivering training packages for midwives and MPS iii) testing the feasibility of implementing the ORAMMA approach care model. Figure 1 illustrates the different phases of the ORAMMA project.

Migrant women were eligible for recruitment into the ORAMMA study if they had been born outside of the host country, they had lived in the host country for less than 5 years, and if they booked for pregnancy care in one of the participating services within the three European countries. Refugees, asylum seekers, spousal migrants, failed asylum seekers and undocumented migrants were all eligible for recruitment. Migrant women within the ORAMMA study ranged in age from 17 to 40 years, were born in one of 19 different countries with 44% of women being born in Syria, included women of all parities with 28% of women being primigravida, 22% having one previous child, 21% already having two children and the remainder already having three or more children. Within each country midwives provided antenatal, intrapartum and postpartum care, with referral to obstetricians should complications be identified. Women received perinatal care according to the local protocols, with care provided where possible by staff who had received ORAMMA cultural competency training. Furthermore, migrant women were matched to a trained MPS who themselves were first- or second-generation migrants, were familiar with the host country and conversant in the host country language. Where possible, women were matched for both first language and ethnicity to an MPS. MPS voluntarily provided practical and emotional support to women throughout the perinatal period.

This paper presents the results from phase 2 of the ORAMMA project (see area outlined in red in Fig. 1); regarding the evaluation of midwives’ compassionate and culturally sensitive maternity care training. Midwives’ experiences of the training as well as caring for migrant women within the ORAMMA project in three European countries are also presented. Results for other ORAMMA project aspects are under preparation [18]. The aim of the study was to evaluate the impact of the compassionate and culturally sensitive maternity care training on midwives’ knowledge, attitude, skills and self-perceived cultural competence.

## Methods

### Study design

A mixed methods approach was undertaken involving both questionnaires and interviews to assess midwives’ training within and experiences of the ORAMMA project.

### Recruitment into the cultural competence training

**Fig. 2:**
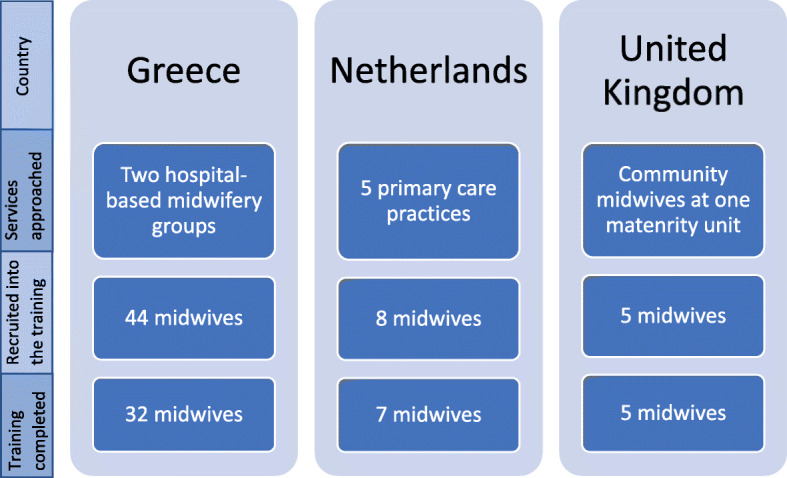
Recruitment of the midwives across the three different countries

A purposive sampling strategy was used. Two hospital-based midwifery groups (Greece), five primary care midwifery practices (Netherlands) and community midwives attached to one maternity unit (UK) were approached (see Fig. 2). Midwives from within these services were identified whose caseloads included migrant women in the Netherlands and UK or who worked in migrant camps or hot spots in Greece. These midwives were invited to participate in the ORAMMA approach and training. A total of 57 midwives were recruited into the training: 8 from the Netherlands, 5 from the UK and 44 from Greece.

### Cultural competence training

The training was informed by evidence regarding cultural competence training [12, 19] as well as systematic reviews of the perinatal experiences of migrants [16] and their healthcare providers. A training manual was developed across all three countries, with training content delivered in accordance with the manual. The training included role play, group discussions, case scenario’s, mini lectures and time to exchange personal experiences of caring for childbearing migrant women. It comprised 3 modules: 1) A background to migration and the issues these women face 2) An overview of maternity care of migrant women and 3) Challenges of and simulated opportunities around effective communication and compassionate, respectful, trauma aware and culturally competent care. Opportunities were provided to improve the three domains of cultural competence; knowledge, attitude and skills. Training was conducted in the native language of each country and was fine-tuned to the local needs according to the different availability of support services and national legal contexts. The training lasted for two full working days in Greece, 3 h with follow-up training of 3 h after 4 months in Netherlands and 4 h in the UK, as deemed acceptable by local participants and their managers in terms of time and availability. Training sessions were organised in January 2018 (Greece and Netherlands) and April 2018 (UK). A one group pre- and post-test design assessed the compassionate and cultural competence training. Participants completed a questionnaire before and immediately after completing the final part of their training. They also completed a brief training evaluation survey.

### Development of the cultural competence assessment questionnaire

Many instruments measuring cultural competence lack validation and standardisation [20, 21]. Our questionnaire was based on the Cultural Competence Questionnaire [19] which incorporates multiple choice questions to test knowledge and the validated Groningen Reflection Ability Scale (GRAS) [22] to test personal reflection ability, adjusted to midwifery related tasks and translated where required. The questionnaire was piloted in the three participating countries. Answers were assigned into culturally competent or incompetent within the three domains: knowledge, attitude and skills. Self-perceived cultural competence (SPCC) was also assessed. Additional File 1 details scores within each domain.

### Interview recruitment and procedure

Midwives who had cared for migrant women within the ORAMMA project were purposively selected to be interviewed to explore their experiences of the project, the training package, working alongside MPS and caring for migrant women within the ORAMMA project. Midwives were approached using various methods across the three countries including email, telephone and face-to-face. All interviewers were female (ES in Greece, LR in the Netherlands and HW and CB in the UK). Participants were aware that the interviewers had been involved with the ORAMMA project and/or training. After providing consent, semi-structured interviews were completed by 5 Greek midwives caring for all 33 ORAMMA participants, 2 Dutch midwives caring for 8 of the 19 ORAMMA participants and 5 British midwives caring for 8 of the 21 ORAMMA participants (Additional File 2 contains the interview guide). For participants’ convenience and to enhance response rates, interviews were conducted in various formats including face-to-face, via telephone or an open-ended online survey. Researchers acted in accordance with Good Clinical Practice standards.

### Data analysis

Questionnaire data around midwives’ knowledge, attitude, skills and self-perceived cultural competence (SPCC) were analysed using SPSS version 24.0. Descriptive statistics summarised participants’ characteristics, cultural competence domain scores and answers to individual questions. Overall domain scores were only calculated if no more than 2 sub-domain items were missing (2 cases were removed from knowledge on medical aspects, 1 from self-perceived cultural competence and 8 from knowledge on interpretation services). Changes were analysed in individual pre and post-test scores using McNemar test for dichotomous data and Wilcoxon signed-rank test for median scores and differences in median domain scores between the three countries using Kruskal-Wallis test.

Interview data from Greece and the Netherlands were translated to English. Audio-recoded and transcribed interviews, alongside field notes were read for familiarity and themes within responses identified using simple content analysis. Data was initially coded at country level by 2 independent researchers within each country and themes identified. Interview data was then integrated across the three countries, verified and agreed by research partners in all countries to develop the overriding themes, which are presented alongside relevant anonymous quotations.

## Results

### Characteristics of midwives

Of the 57 midwives recruited to the ORAMMA training, 35 completed both the pre- and post-training questionnaires (23 of 44 Greek, 7 of 8 Dutch and 5 of 5 British midwives) and were included within the analysis. The loss to follow up in Greece was due to midwives being unable to spend two days away from their job to complete the training. For the same reasons, shorter trainings were undertaken in the Netherlands and UK.

Table 1 provides baseline characteristics for the 35 midwives completing the training. All midwives were female and the majority were native citizens, experienced with supporting pregnant migrants. Over half had practised for more than 5 years. Midwives in all countries had comparable, moderate CC GRAS-scores, indicating moderate reflection ability.

**Table 1:** Baseline characteristics of midwives receiving training on culturally sensitive maternity care (*n* = 35)

	All (***n*** = 35)	Greece (***n*** = 23)	Netherlands (***n*** = 7)	United Kingdom (***n*** = 5)
**Age** (range)	37.9 (24–60)	37.9 (24–60)	33.9 (25–54)	43.6 (27–54)
**Migration status** % migrant	2.9% (1/34)	4.5% (1/22)	0% (0/7)	0% (0/5)
**CC GRAS-score** (median [Inter quartile range]) scale 10–50	35.00 [30.0,37.0]	34.0 [29.0,38.0]	36.0 [30.0,37.0]	35.0 [31.0,37.0]
**Number of pregnant migrants supported in last 5 years**				
None	0.0% (0/35)	0% (0/23)	0% (0/7)	0% (0/5)
1–10 women	2.9% (1/35)	0% (0/23)	14.3% (1/7)	0% (0/5)
> 10 women	97.1% (34/35)	100% (23/23)	85.7% (6/7)	100% (5/5)
**Experience with language barriers in last 6 months**				
None	8.6% (3/35)	13.1% (3/23)	0% (0/7)	0% (0/5)
1–5 women	20.0% (7/35)	30.4% (7/23)	0% (0/7)	0% (0/5)
6–10 women	8.6% (3/35)	8.7% (2/23)	14.3% (1/7)	0% (0/5)
> 10 women	62.8% (22/35)	47.8% (11/23)	85.7% (6/7)	100% (5/5)
**Experience as a midwife**				
< 1 year	Unknown*	Unknown*	0% (0/7)	0% (0/5)
1–5 years			57.1% (4/7)	0% (0/5)
6–10 years			0% (0/7)	40% (2/5)
> 10 years			42.9% (3/7)	60% (3/5)

* Questions missing from the translations in the questionnaire for that country

*CC-GRAS* Cultural Competence Groningen Reflection Ability Scale. The Groningen Reflection Ability Scale (GRAS) is a validated scale that measures participants’ general ability of personal reflection (Aukes et al. 2007) [19]. Seeleman et al. (2014) [18] adjusted the GRAS to assess reflection ability as part of cultural competence. We shortened the adjusted score into 10 items. All 10 items score 1 to 5 and together make up the cultural competence (CC) GRAS-score between 10 and 50, with higher scores indicating higher reflection ability

### Domain score results

**Fig. 3:**
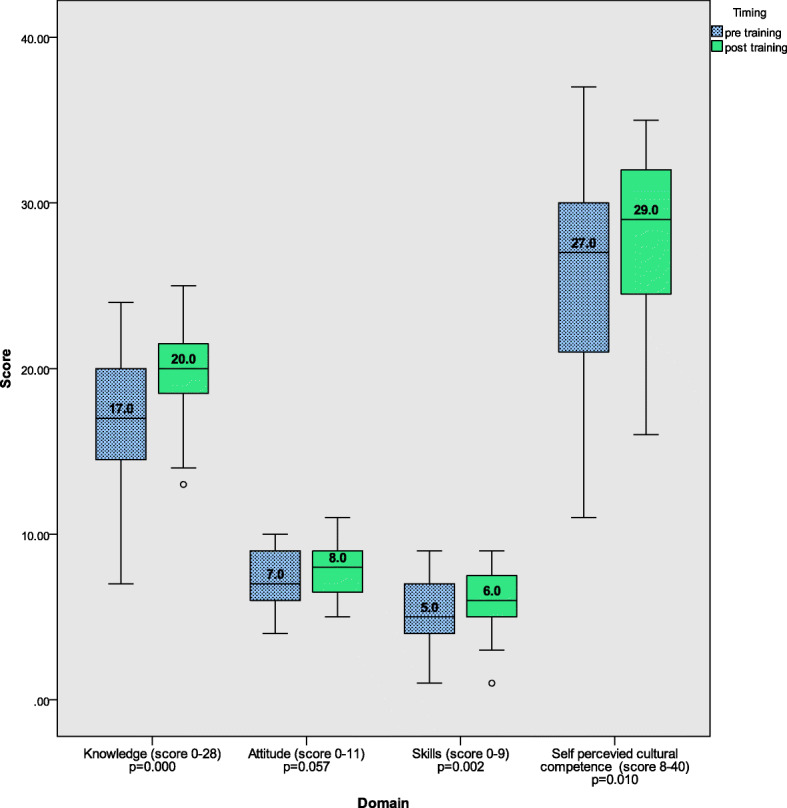
Overall median pre and post-test scores within each domain (*N* = 35) Differences between median pre- and post-score tested using Wilcoxon signed-rank test

Figure 3 shows the overall scores in all midwives for the three domains of cultural competence and SPCC before and after training on culturally sensitive maternity care and Table 2 in individual countries.

**Table 2:** Median scores for knowledge, attitude, skills and self-perceived cultural competence within each country

	Greece (***N*** = 23)			Netherlands (***N*** = 7)			UK (***N*** = 5)			Between Country differences	
	Pre	Post	***p value***	Pre	Post	***p value***	Pre	Post	***p value***	Pre	Post
**Knowledge** (score 0–28)											
Median [IQR]	16 [14–18]	19 [17–20]	0.001**	20 [17–21]	22 [20–22]	0.026*	20 [16.5–22]	23 [21–24]	0.041*	0.018*	0.001**
**Attitude** (score 0–11)											
Median [IQR]	7 [6–9]	7 [6–9]	0.942	7 [6–10]	9 [7–10]	0.221	8 [7.5–9]	11 [9.5–11]	0.042*	0.472	0.001**
**Skills** (score 0–9)											
Median [IQR]	6 [4–7]	5 [4–8]	0.156	4 [4–6]	6 [5–7]	0.047*	4 [3–6]	6 [5–7.5]	0.039*	0.450	0.787
**Self-perceived cultural confidence (SPCC)** (score 8–40)											
Median [IQR]	26 [19.5–30.5]	28 [22–33]	0.057	25 [21–30]	28 [24–32]	0.234	30 [28–30.5]	32 [30.5–33.5]	0.068	0.259	0.169

Differences between median pre- and post-test score within each country tested using Wilcoxon signed-rank test

Differences between pre- and post-test scores between countries tested using Kruskall Wallis test

*IQR* interquartile range

* *p* < 0.05, ** *p* < 0.01, ***p < 0.001

#### Knowledge

The median knowledge score significantly improved overall and in each individual country (Median pre-test to post-test score being 17 to 20 out of a total score of 28 overall (*p* < 0.001, Fig. 3), 16 to 19 (*p* = 0.001) in Greece, 20 to 22 (*p* = 0.026) in Netherlands and 20 to 23 (*p* = 0.041) in UK, Table 2)***.*** Differences were noted in pre and post-test knowledge scores between countries (*p* = 0.018 and 0.001 respectively, Table 2), with scores suggesting higher knowledge in the Netherlands and UK than in Greece.

For sub-domains of knowledge (Table 3), a significant increase of knowledge on medical aspects was seen in the total group (*p* = 0.000) and in Greek and Dutch midwives (*p* = 0.007 and *p* = 0.041 respectively). No statistically significant change was noted in any other knowledge sub-domain.

**Table 3:** Pre and post-test scores within each sub-domain for each country

	Greece (***N*** = 23)			Netherlands (***N*** = 7)			UK (***N*** = 5)			Overall pre-post
	Pre	Post)	***p value***	Pre	Post	***p value***	Pre	Post	***p*** value	***P*** value
**Knowledge on: (Median [IQR])** ^**a**^										
Medical aspects (max score 12)	6.5 [5–8.25]	9.0 [7–10]	0.007**	9 [6–11]	10 [10–11]	0.041*	8 [4–10]	11 [10–11.5]	*0.068*	0.000***
Interpretation services (max score 5)	1.5 [1–2]	2 [1–2.5]	*0.454*	1 [1–2]	2 [1–2]	*0.157*	2.5 [1.3–4.5]	2.5 [2–3]	*0.854*	*0.404*
National legislation (max score 3)	2 [2–3]	2 [2–3]	*0.480*	2 [2–3]	2 [2–3]	*0.564*	2 [2–3]	3 [2.5–3]	*0.157*	*0.405*
Ethnic minority patients (max score 8)	7 [6–7]	7 [6–7]	*0.435*	7 [6–8]	7 [7–8]	*0.157*	8 [7–8]	7 [6–8]	*0.180*	*0.569*
**Attitude - (Yes %)** ^**b**^										
I respect her choice to become pregnant	52.2%	65.2%	*0.250*	42.9%	57.1%	*1.000*	40.0%	100%	*0.250*	0.016*
I understand why scheduled appointments are hard	56.5%	73.9%	*0.125*	85.7%	71.4%	*1.000*	40.0%	100%	*0.250*	*0.070*
I feel irritated when she fails to attend twice ^c^	4.3%	4.3%	*1.000*	71.4%	28.6%	*0.250*	0%	0%	*–*	*0.250*
I cannot understand why she would bring a child into the world in her situation ^c^	0%	0%	*–*	14.3%	14.3%	*1.000*	0%	0%	*–*	*1.000*
I feel empathy for her	4.3%	8.7%	*1.000*	42.9%	57.1%	*1.000*	60.0%	100%	*0.500*	*0.219*
I’m worried about her	73.9%	82.6%	*0.625*	100%	100%	*–*	80.0%	100%	*1.000*	*0.375*
I’m worried about her child	56.5%	52.2%	*1.000*	100%	85.7%	*1.000*	80.0%	100%	*1.000*	*1.000*
I feel desperate as I have no idea how to help ^c^	4.3%	4.3%	*1.000*	28.6%	0%	*0.500*	0%	0%	*–*	*0.625*
I’m glad I will be able to help her	56.5%	52.2%	*1.000*	28.6%	85.7%	*0.123*	80.0%	100%	*1.000*	*0.344*
I’m considering informing child protection ^c^	39.1%	47.8%	*0.625*	0%	0%	*–*	20.0%	20.0%	*1.000*	*0.625*
I feel I need to consult more experienced colleagues	69.6%	43.5%	0.031*	42.9%	42.9%	*1.000*	60.0%	60.0%	*1.000*	*0.109*
**Skills (Yes - %)** ^**b**^										
I will end my care as usual in my country ^c^	8.7%	8.7%	*1.000*	57.1%	42.9%	*1.000*	20.0%	20.0%	*1.000*	*1.000*
I will approach different organisations for support	78.3%	73.9%	*1.000*	57.1%	85.7%	*0.500*	20.0%	60.0%	*0.500*	*0.453*
Discuss pros and cons of government accommodation	43.5%	47.8%	*1.000*	57.1%	71.4%	*1.000*	60.0%	60.0%	*1.000*	*0.754*
Write to the government requesting stay in current shelter until postnatal visits complete	26.1%	21.7%	*1.000*	0%	14.3%	*1.000*	20.0%	20.0%	*1.000*	*1.000*
Concerns over victim of sexual violence	52.2%	56.5%	*1.000*	42.9%	57.1%	*1.000*	20.0%	60.0%	*0.500*	*0.289*
Inform General Practitioner (GP) and health visitor over medical and mental health risk concerns	56.5%	69.6%	*0.453*	71.4%	100%	*0.500*	80.0%	100%	*1.000*	*0.109*
Concerns over Female Genital Mutilation (FGM)	13%	30.4%	*0.125*	42.9%	42.9%	*1.000*	0%	40%	*0.500*	0.031*
No partner so contraception not discussed ^c^	4.3%	4.3%	*1.000*	14.3%	14.3%	*1.000*	0%	0%	*–*	*1.000*
Inform of relevant social and other care	82.6%	87.0%	*1.000*	57.1%	100%	*0.250*	60%	100%	*0.500*	*0.070*
**SPCC (each sub-domain scored from 1 to 5) (Median [IQR])** ^**a**^										
**I feel capable of providing adequate care in relation to:** Communication with a language barrier	3 [2.75–3]	4 [3–4]	*0.090*	3 [3–4]	4 [3–4]	*0.157*	4 [4–4.5]	4 [4–5]	*0.317*	0.025*
handling cultural differences	4 [3–5]	4 [3–5]	*0.180*	3 [3–4]	4 [3–4]	0.046*	4 [3–4]	4 [4–4]	*0.157*	0.007**
discuss (sexual) violence	3 [2.5–4]	3 [3–4]	*0.351*	3 [2–4]	4 [3–4]	0.046*	4 [3–4]	3 [3–4]	*0.564*	*0.788*
ask about FGM	3 [2–4]	3 [2–4]	*0.412*	4 [3–4]	4 [3.5–4]	*0.414*	4 [4–5]	4 [4–4]	*0.157*	*0.446*
manage consequences of FGM	3 [2–4]	3.5 [2–4]	*0.414*	4 [3–4]	4 [3–4]	*1.000*	4 [3–4]	4 [4–4]	*0.157*	*0.276*
provide health promotion to migrants	4 [3–5]	4 [3.75–5]	*0.102*	3 [2–4]	3 [3–4]	*0.480*	4 [3.5–4]	4 [4–4.5]	*0.157*	0.046*
legal and procedural aspects around migration status	2 [1–3]	3 [2–3]	0.013*	1 [1–2]	2 [1–3]	*0.096*	2 [1.5–2.5]	4 [3–4]	0.038*	0.000***
refer to social care	4 [3–4]	4 [4–5]	0.019*	3 [3–5]	4 [4–4]	*0.317*	4 [4–4.5]	4 [4–5]	*0.317*	0.007**

^a^ - domains negatively scoring - so a decrease in score is desired

^b^ - McNemar Test used for dichotomous variables to test for differences between pre- and post-test proportions within each country and overall

^c^ - Differences between median pre- and post-score within each country and overall tested using Wilcoxon signed-rank test

*IQR* Interquartile range

* *p* < 0.05, ** *p* < 0.01, ***p < 0.001

#### Attitude

No significant improvement was seen in the overall median attitude score (*p* = 0.057, Fig. 3). Only in the UK was a significant improvement seen in the median pre-test score of 8 to post-test score of 11 (*p* = 0.042, Table 2). Median attitude pre-test scores were not significantly different between the countries, however median post-test score was significantly different, with Greece having the lowest and the UK the highest post-test scores (*p* = 0.001, Table 2).

Analysis on the individual questions comprising the attitude score (Table 3) revealed significantly more midwives stated they respected and understood the choice of migrant women to become pregnant under difficult circumstances (48.6% before training, 68.6% after training, *p* = 0.016). No other individual attitude scores significantly differed. However, within country analysis showed fewer Greek midwives felt the need to consult colleagues more experienced in caring for childbearing migrants post training (69.6% pre training, 43.5% post training, *p* = 0.031).

#### Skills

An improvement of the skills score was seen in the Netherlands (*p* = 0.047), UK (*p* = 0.039), and total group of midwives (*p* = 0.002, Fig. 3 and Table 2).

#### Self-perceived cultural competence (SPCC)

An improvement in median SPCC pre and post-test was seen in the overall group of midwives (*p* = 0.010, Fig. 3), but did not reach significance in any individual country (Table 2).

In-depth analysis of the sub-domains of SPCC showed an increase in midwives self-perceived capability to communicate with migrants when there was a language barrier, to handle cultural differences, to provide health promotion to migrant women, in understanding legal and procedural aspects around migration status and to refer to social care (*p* < 0.05).

### Experiences of training

When assessing midwives’ experiences of the training, three themes emerged; “appropriate and applicable”, “made a difference” and “training gaps”.

#### Appropriate and applicable

Most midwives were generally positive about the training; feeling it was relevant to their practice and would influence the care they provided to recently arrived migrant women.

*"I am going to use this to try to improve how I deliver care and to put the ladies* [migrants] *in a position of power" (UK5)*

*"The training was easy to put in practice" (Netherlands2)*

*'I am now more aware of personal gaps in knowledge and skills concerning care for pregnant migrants'. (Netherlands3)*

#### Made a difference

Midwives felt they gained a better understanding of migrant women’s behaviour such as reasons for non-attendance at antenatal care and so were better prepared for putting migrant women at ease within an appointment. Aspects of training particularly appreciated by midwives in Greece were how to approach vulnerable groups from different cultural backgrounds with empathy, in the UK the cultural competency and trauma-aware care components and in the Netherlands the use of an actress to undertake roleplay.

*“The* [migrant] *actress was really good and I could easily put this in practice” (Netherlands1)*

*“To learn about their culture, the importance of their habits ... to know and understand them and to see them with empathy” (Greece2)*

#### Training gaps

All midwives wished for more in-depth training on certain aspects; however these varied between the countries. Greek midwives wanted more focus on living conditions of migrant women to ensure understanding and appropriate advice could be provided.

*“Most of these women were not happy living in camps and were anticipating to be relocated to West European countries – which seemed to be their main concern, thus they were not focusing on the perinatal care, but rather on their relocation possibilities” (Greece3)*

In the UK midwives wanted;

"*More information re legal status and financial help. Where to signpost women to*" *(UK4)*

All midwives wanted communication and cultural competence training to be provided to all healthcare workers and felt that course accreditation would assist this. Dutch midwives also recognised a need to develop evidence-based protocols around migrant maternity care alongside providing training.

*"Access to these trainings and good quality protocols would improve care" (Netherlands1)*

### ORAMMA project experiences

The semi-structured interviews exploring midwives experiences of caring for migrant women within the ORAMMA project identified three themes; “supportive care”, “working alongside peer supporters” and “challenges faced”.

#### Supportive care

Midwives were generally enthusiastic about the ORAMMA project and wanted the project to continue. They felt the initiative provided a specialised approach for caring for migrant women including empathy, understanding and respect. In Greece, where women were mainly accommodated in camps, it was particularly viewed as an efficient link between primary and secondary healthcare.

*"The project is interesting and has the ability to improve the care that pregnant migrants receive" (Netherlands1)*

*“That was a start; I just wish it would continue to be a program just like that … As ORAMMA we had a very supportive role between structures and secondary healthcare. We were a connecting link in facilitating their contact with secondary healthcare, where the camps’ system was lagging behind” (Greece1)*

Midwives believed the ORAMMA project’s MPS provided migrant women with continuity as well as individual help and social support that they as professionals could not offer within the maternity system, such as showing the women places that could provide baby equipment.

A midwife in the Netherlands also voiced that she felt the women themselves liked the project as she found;

*"It was easy to recruit refugee women for this project. The leaflet was always on my desk, and was translated into 5 different languages. Often, women took this leaflet themselves" (Netherlands2)*.

#### Working alongside peer supporters

Due to the voluntary nature of the role not all MPS attended healthcare appointments with the woman. Dutch midwives could contact MPS through email and telephone conversations to improve communication with the migrant woman. Where MPS had attended with women, some midwives felt they improved communication, for example enabling the midwives to fully explain the clinical procedures offered and that woman themselves were more confident to ask questions or express concerns. Women also had improved knowledge about their new country and its healthcare system. Being able to match women with someone that spoke the same language was felt to be crucial for these improvements.

*"The supporter attended appointments at the hospital, community midwife clinics and at the family's home. She offered flexibility and some consistency, in difficult circumstances. This was so valuable for this family's experience of maternity care" (UK3)*

*“We could not be efficient without the maternity peer supporters. We could not get their health record, nor could we provide services in sign language. Without them there would be no communication” (Greece3)*

However, it was clear that successful support was dependent on the relationship that developed between the woman and the MPS. Some midwives felt that women shared more when the MPS wasn’t present.

*"The woman also shared much more with me through language line* [telephone interpreters] *when the peer support wasn't available. It was as if she didn't trust the peer support." (UK2)*

In Greece one midwife felt the use of MPS could cause confusion if their role and responsibilities were not clearly defined, especially for women originating from countries with different care systems for example traditional rather than professional midwives. Dutch midwives however felt roles were clear with MPS helping refer migrant women to healthcare providers if they were experiencing any problems.

*"I just believe that the MPS more easily refers the woman to us and I trusted the MPS that she knew her role" (Netherlands2)*

#### Challenges faced

Midwives faced some challenges in caring for migrant women within the ORAMMA project. This included difficulty in maintaining contact with women throughout the perinatal period and women’s struggles to attend appointments due to issues with distances to travel, childcare and relocation. Women often arrived too early or late for appointments, making it difficult to provide the required care. Some women also only visited healthcare providers if they had a problem.

*“Unless they have a problem, which these women usually do not have, either with breastfeeding or generally with their postpartum period, they will not seek out a health professional, they do not have it in their routine, as we do. If they don't feel something bad, they won't do it.” (Greece4)*

Issues specific to the ORAMMA project, including that while one MPS was bilingual they were unfamiliar with the host country’s maternity system which hindered the support she could provide. Should ORAMMA continue, midwives wanted the MPS role to be paid, to start as early as possible during pregnancy and for an introductory meeting between midwives and MPS to establish a working relationship with clear expectations.

*"I would like to know the MPS better and to make some clear agreements with them. I don’t want to take advantage of them so I want to know what I can ask of them." (Netherlands2)*

Midwives wished for longer appointment times as *“You need more time to identify the psychosocial situation* [of migrant women]*” (Netherlands1).* There was also recognition that even with an MPS more time was needed in the presence of a language barrier. *“A midwife has limited time gaining history with no time weighting for second language” (UK2).* However, there was recognition this would require additional government funding. Midwives also desired continuity of midwife care for migrant women, including during labour for low risk pregnancies.

## Discussion

Compassionate and culturally sensitive maternity care training resulted in significantly increased knowledge, self-perceived cultural competence and skills scores among midwives. The ORAMMA training was generally well received by midwives. They also appreciated the support and enhanced communication with migrant women through the ORAMMA approach including integrated multidisciplinary care, cultural competence training for health providers and the support of an MPS, although this was not without its challenges.

The positive association between training and increased cultural competence domains of knowledge and skills is in line with previous studies [12–14]. It is recognised that attitudes need time to change [10]; however training under 8 h has previously been shown to positively effect healthcare providers’ attitudes [12]. Pre-test attitude scores were high within our sample, which could be due to selection bias as participating midwives were purposefully recruited due to their involvement in caring for migrant women. Despite having the highest pre-test scores, midwives in the UK showed further improvements of the attitude score. The lack of improvement in this domain across all countries may therefore reflect local differences in the actual training provided, although prior agreements on the content was in place.

Greek midwives having lower knowledge scores than the other sites could in part be explained by a later influx of refugees to Greece. Migrants arriving into Greece in 2015 and 2016 were the country’s first experience of mass migration from Africa and the Middle East. In the 2001 census only 0.69% of the Greek population were African, Asian or South American; with immigration prior to this being primarily from Eastern and Central Europe [23, 24]. In contrast the UK and Netherlands have experienced an influx of these migrants for many decades. This may also explain the decrease in Greek midwives feeling the need to consult colleagues more experienced in caring for childbearing migrants post training, as their knowledge around the care of migrants increased.

While midwives mainly viewed MPS as an invaluable source of support for migrant women, this was influenced by the quality of the relationship built between the woman and MPS. Others too have found women may negatively evaluate peer support for example if they feel their problems are belittled [25]. This may in part be due to role expectations. As peer support does not have one universal agreed definition, it is essential that each project clearly specified the services they can provide [26].

### Strengths and limitations

We believe this is the first quantitative study assessing the effect of training on midwives’ cultural competence and whether country differences impact upon training effectiveness. A clear framework of cultural competence [11] was utilised to provide a comprehensive, detailed description of participants and training interventions to facilitate correct intervention replication; data that is lacking in most previous studies [27]. Furthermore within the interviews, themes were similar across the three countries indicating data saturation.

There were however limitations within the study. Midwives were unequally divided over the countries, with Greece contributing 66% of the total group and therefore having a large impact on overall scores. The length of training varied between the countries. Greece had a large loss to follow-up due to midwives being unable to spend two days away from their job to complete the training. For the same reasons, shorter trainings were undertaken in the Netherlands and UK. There was also some variation in the role of healthcare professionals and other organisations across countries. Findings within each sub-domain are therefore provided separately for each country. Heterogeneity within the training sessions and the absence of a control group might have weakened the external validity. However, one group, pre- and post-test study designs are widely and reliably used to assess the impact of training [28]. The varied settings is also a strength of this study as it reflects the reality of any future implementation in which countries’ training sessions will differ according to the contextual background and differences in the workforce experiences and motivation in working with migrants.

Cultural competency assessments were not previously available for the maternity care environment. In adapting previous measures, the knowledge questions may have not been challenging enough to assess further improvement of the scores. The score improvement of all domains may underestimate the actual learning effect of the training as some participants may be transformed from being an “unconscious incompetent” to “conscious incompetent” [29], as reflected by one Dutch midwife’s comments who verbalised they had become more aware of their personal gaps in knowledge.

Our questionnaire evaluated cultural competence by using a knowledge test and questions around case scenarios to determine intended behaviour and attitudes [19]. However, self-assessment of attitude and skills presents a risk for social desirability bias [30]. Objective assessment of attitude and skills through videotaped clinical encounters or objective structured clinical examinations is desirable [31]. It is recognised that we tested whether midwives learnt what was taught and further evaluation is required regarding implementation of acquired knowledge into practice and any impact on clinical outcomes [32, 33]. However, testing the achievement of learning objectives in this study is the first step in educational programme evaluation and should not be overlooked.

### Implications for practice

While the ORAMMA training with its emphasis on providing knowledge based on attitudes and skills was ideal to address the diverse needs of staff across the different countries, it clearly shows country specific training evaluation is important in any future multi-country implementation.

Self-perceived competence significantly increased in the sub-domain of legal and procedural aspects of migrant status however this complex issue was identified as an area which midwives would still like more detailed information provision in future trainings.

In summary, providing culturally competent care is essential to meet the needs of the growing migrant populations in Europe and healthcare providers require training to deliver this care. This novel international study provides empirical evidence that the ORAMMA compassionate and culturally sensitive maternity care training improves midwives’ knowledge, skills and self-perceived cultural competence. Future research assessing the transfer of gained cultural competence knowledge into practice and the sustainability of changes would be beneficial in a range of healthcare providers.

## Conclusions

This study provides important primary information on the effectiveness of the ORAMMA cultural competence training in enhancing midwives’ knowledge, skills and self-perceived competence. The training was generally well received by the midwives, who felt it would influence the care they would provide to recently arrived migrant women. Midwives across the three European countries also appreciated the support and enhanced communication provided by the ORAMMA care model. However further investigation on the complementary role of maternity peer supporters alongside research into the impact of the ORAMMA approach and training on clinical outcomes, is merited.

## Supplementary Information

**Additional file 1.**

**Additional file 2.**

## Data Availability

The datasets used and/or analysed during the current study are available from the corresponding author on reasonable request.

## Abbreviations

CC-GRAS: Cultural Competence Groningen Reflection Ability Scale
FGM: Female Genital Mutilation
GRAS: Groningen Reflection Ability Scale (GRAS)
IQR: Inter Quartile Range
MPS: Maternity Peer Supporter
ORAMMA: Operational Refugee and Migrant Maternal Approach
SPCC: Self Perceived Cultural Competence
UK: United Kingdom
WHO: World Health Organization
