# Supplementary material for: Midwives’ experiences of cultural competency training and providing perinatal care for migrant women a mixed methods study: Operational Refugee and Migrant Maternal Approach (ORAMMA) project
Source: BMC Pregnancy Childbirth. 2021 Apr 29;21:340. doi: 10.1186/s12884-021-03799-1 (PMC8082812; doi:10.1186/s12884-021-03799-1)
Supplement: Supplementary file 1 — Additional file 1. [file 12884_2021_3799_MOESM1_ESM.pdf]

Additional File 2. Number of items, type of assessment and total possible minimum and maximum scores for each domain and subdomain (knowledge, attitude, skills) and self-perceived cultural competence

Item	Number of items	Type of assessment	Score (minimum-maximum)
**Knowledge: Possible scores 0-28**			
Knowledge on medical aspects	12	Likert-scale question with 3 response options: more often, equally often, less often	0-12
Knowledge on interpretation services	5	Likert-scale question with 4 response options: never, most times not, most times, always appropriate	0-5
Knowledge on national legislation	3	Yes/No-question with 3 categories: correct, incorrect, I don't know	0-3
Knowledge on ethnic minority patients	8	Yes/No-question with 3 categories: correct, incorrect, I don't know	0-8
**Attitude: Possible scores 0-11**			
General attitude towards ethnic minority patients	11	Multiple-answer multiple choice question	0-11
**Skills: Possible scores 0-9**			
Provision of culturally sensitive care	9	Multiple-answer multiple choice question	0-9
**Self-perceived cultural competence: Possible scores 8-40**			
Self-perceived cultural competence on specific aspects of care for pregnant migrants	8	Likert-scale question with 5 response options: not at all capable (1) to very capable (5)	8-40
