# Supplementary material for: Midwives’ experiences of cultural competency training and providing perinatal care for migrant women a mixed methods study: Operational Refugee and Migrant Maternal Approach (ORAMMA) project
Source: BMC Pregnancy Childbirth. 2021 Apr 29;21:340. doi: 10.1186/s12884-021-03799-1 (PMC8082812; doi:10.1186/s12884-021-03799-1)
Supplement: Supplementary file 2 — Additional file 2. [file 12884_2021_3799_MOESM2_ESM.pdf]

Additional File 1. Focus group/ interview schedule for Healthcare professionals

We are very grateful to you all for sparing time to take part in this research to talk about your experiences of the ORAMMA project. The discussion will be recorded and then written up word for word to make it easier for the researchers to evaluate what has been said afterwards. Your name or any other identifying information will not be included in this transcript (we will give you a code name), so anything you say will not be able to be linked back to you.

Today I want to concentrate on how you felt about taking part in the ORAMMA project. There are no right or wrong opinions; I would like you to feel comfortable saying what you really think and how you really feel.

- What are your experiences of the ORAMMA project?
- What did you think about the ORAMMA training package?
    - Were there any parts that were particularly useful?
    - Were there any parts that weren't particularly useful?
    - Is there anything else you wish had been covered in the training?

- What were your experiences of working alongside maternity peer supporters?
    - What were the benefits of working alongside a maternity peer supporters?
    - What were the challenges of working alongside a maternity peer supporters?

- What are your experiences of caring for migrant / refugee women?
    - Are there any further resources you would like to see available to help you care for these women?

- Do you have any further comments you would like to make about the ORAMMA project?

Thank you again for taking part, we really appreciate it.
